# Analysis of the role of human leukocyte antigen class-I genes to understand the etiopathology of schizophrenia

**DOI:** 10.4103/0019-5545.43625

**Published:** 2008

**Authors:** Bisu Singh, Sikta Banerjee, Nirmal K. Bera, Chitta R. Nayak, Tapas K. Chaudhuri

**Affiliations:** Department of Zoology, Cellular Immunology Laboratory, University of North Bengal, Siliguri 734 430, West Bengal, India; 1 Department of Psychiatry, North Bengal Medical College and Hospital, Siliguri - 734 430, West Bengal, India; 2 Computer Centre, University of North Bengal, Siliguri - 734 430, West Bengal, India

**Keywords:** Etiology, human leukocyte antigen, schizophrenia

## Abstract

**Background:**

Schizophrenia is the paradigmatic illness of psychiatry. The involvement of immunological and immunopathological mechanisms in the etiopathogenesis of schizophrenia has been a matter of research, with recently increasing effort.

**Aims:**

In this study, we investigated the incidence of human leukocyte antigen (HLA) Class I antigens to understand the role of HLA genes in schizophrenia.

**Materials and Methods:**

India born schizophrenic patients in and around Siliguri who attended outpatient department (OPD) of Department of Psychiatry, North Bengal Medical College and Hospital were considered for the present study. After the longitudinal follow up, 50 patients were enrolled for the study. The same number of age, sex and ethnically matched healthy subjects were considered as control. Low resolution polymerase chain reaction-sequence specific primer method was applied for typing the HLA antigens.

**Statistics:**

The phenotype frequencies were calculated by direct count. χ^2^ test was done to compare the frequency of each antigen among the patients and control group and it was followed by Fisher's exact test. Relative risk was estimated by using Haldane's method.

**Results:**

The result showed that some of the HLA antigens are associated with the schizophrenia and significant increase were observed for HLA A*03 antigen along with the significant decrease for HLA A*25, A*31 and HLA B*51.

**Conclusions:**

The study provides the evidence for the possible existence of susceptibility locus for schizophrenia within the HLA region. This preliminary observation may help to understand the etiological basis of this disorder and the study may further strengthen the HLA antigens as the marker for schizophrenia.

## INTRODUCTION

Schizophrenia is a severely debilitating neuropsychiatric disorder characterized by “disturbances of thought, auditory hallucinations and multiple delusions”.[1] It affects 1% of the worldwide population.[2] The essential biological pathology of schizophrenia ia partially understood till to date.[3] However, there is substantial evidence to indicate a major genetic component.[4][5] Different chromosomes have been pinpointed as harbouring genes involved in the pathogenesis of schizophrenia.[6] A susceptibility locus has been identified on chromosome 6.[7][8] Several researches have also found evidence for schizophrenia vulnerability genes on chromosome 6p close to the HLA genetic region by linkage analysis.[9] HLA and schizophrenia was first reviewed by Mc Guffin (1979),[10] who commented that the MHC was a logical place in which to search for genetic markers for schizophrenia because schizophrenia was similar to diseases for which HLA association had been established in that it was familial, had an imperfectly understood etiology, and had a postulated autoimmune pathogenesis.[11]

The first HLA association study of schizophrenia was reported by Cazzullo *et al.*, in 1974.[12] More than 60 association studies have been reported since then.[13] The details of past association studies is given in Table 1.

**Table 1:** HLA association studies of schizophrenia- Class I (A, B and C) antigens*†

Investigator, year‡	Ethnicity Diagnosis (number)	Patient subjects Origin (number)	Comparison subjects	Result (comment)
Cazzullo *et al.*, 1974	Caucasian	Feignner (52)	Population (386)	no association
Eberhard *et al.*, 1975	Caucasian	Bleuler (47)	-(1263)	A9 (RR=2.9)
Ivanyi *et al.*, 1976	Caucasian	-(148)	Population (1200)	A28(RR=3.4)
Smeraldi *et al.*, 1976a	Caucasian	Feighner (70)	Popualtion (386)	No association
Smeraldi *et al.*, 1976b	Caucasian	Feighner (144)	Population (386)	A10 (RR=0.4)
Julien *et al.*, 1977	Caucasian	-(65)	Population (250)	A9 (RR=2.5)
Ivanyi *et al.*, 1977	Caucasian	-(40)	Population (438)	Cw4 with paranoid schizophrenia (RR=3.7)
			Population (1200)	B18 with paranoid schizophrenia (RR=3.4)
Bennahum *et al.*,1977	Caucasian	Feighner (38)	-(102)	No association
Kyner *et al.*, 1978	Caucasian	Feighner (20)	-(67)	No association
Ivanyi *et al.*,1978	Caucasian	-(200)	Population (1200)	A28(RR=3.0)
Mc Guffin *et al.*, 1978	Caucasian	ICD-9 (80)	Blood donors(458)	No association
Perris *et al.*, 1979	Caucasian	-(50)	Blood donors (449)	No association
Crowe *et al.*, 1979	Caucasian	Feighner (45)	Population(1263)	Aw 10 (A26 subtype) with hebephrenia (RR=6.6)
Luchins *et al.*, 1980	Caucasian	RDC(38)	Published data (743)	No association
	African-USA	RDC(92)	Published data (563)	A2 (RR=2.3)
Gattaz and Beckmann,1980	Caucasian	Feighner (100)	-(472)	B27 with poor prognosis patients
Mendlewicz *et al.*, 1981	Caucasian	Feighner (64)	Blood donors (113)	No association
Asaka *et al.*, 1981	Japanese	-(136)	Blood donors (187)	A9(Aw24 subtype)(RR=2.0) A10(A26 subtype)(RR=1.9)
Goudemand *et al.*, 1981	Caucasian	-(51)	Blood donors (94)	No association
Singer *et al.*, 1982	Caucasian	-(75)	Blood donors (184)	No association
Ivanyi *et al.*, 1983	Caucasian	Feighner (62)	-(1018)	No association
Rosler *et al.*, 1980	Caucasian	Feighner (107)	Blood donors (600)	A28 (RR=3.1)
Miyanaga *et al.*, 1984	Japanese	DSM-III (77)	Blood donors (1252)	No association
Rudduck *et al.* 1984a	Caucasian	DSM-III (100)	Blood donors (919)	No association
Rudduck *et al.* 1984b	Caucasian	DSM-III (116)	Blood donors (919)	No association
Adler *et al.*, 1985	Caucasian	RDC (14)	-(365)	No association
Amar *et al.*, 1988	Jewish	-(32)	-(151)	No association
Metzer *et al.*, 1988	Caucasian	DSM-III (53)	Blood donors (114)	No association
Alexander *et al.*, 1990	Caucasian	DSM-III (55)	Published data (1029)	No association
DiMichele *et al.* 1990	Caucasian	DSM-III (36)	-(500)	No association
Campion *et al.*, 1991	Caucasian	DSM-III (107)	Relatives (174)	No association
Wright *et al.*, 1995	Caucasian	DSM-III-R (93)	Screened controls (141)	A9 (RR=1.94)
				A24 subspecificity Of A9 (RR=2.76)
Blackwood *et al.*, 1996	Caucasian	RDC&DSM-III-R (107)	-(133)	B35(corrected P=0.004, RR=0.06)
				Cw5 (corrected P=0.05, RR=0.38)
		Blood donors (264)	B35	
Ozcan *et al.*, 1996	Caucasian	-(75)	-(3731)	No association
Jocobsen *et al.*, 1998	Caucasian	DSM-IV(28 children)	Population controls (51)	No association
Gibson *et al.*, 1999	Caucasian	DSM-III-R (256)	Blood donors (261)	No association
Debnath *et al.*, 2005	Indian (Bengalee)	DSM-IV-TR (50)	Blood donors (100)	A3 (RR=5.66)
*Total studies*	*Total patients per ethnic group*	*Total controls per group§*	*Associations reported more than once*	
35 serotyping studies	Caucasian 3146 (including 28 children	7802 unknown	4 studies: A9 or A24 subspecificity of A9	
1 genotyping study	Japanese 213	4895 blood donors	3 studies: A28	
	African-USA 92	4788 population	3 studies: A10	
	Jewish 32	2335 published data		
	174 relatives			
	141 screened controls			

* Table based on data from Nimgaonkar *et al.*, (1992), Hawi et *et al.*, (1999), Index Medicus, MEDLINE and EMBASE searches from 1974 to 2000, and personal communications

† RR=relative risk when significant association remains after correction for multiple comparisons; n=number of schizophrenic patients or number of controls; diagnostic criteria utilized in the above studies are those of Feighner *et al.*, (1972) and Bleuler (1950), the International Classification of Diseases 9 (WHO,1978), the Diagnostic and Statistical Manual III, III-R and IV (American Psychiatric Association, 1980, 1987, 1994) and the Research Diagnostic Criteria of Spitzer *et al.*, 1978)

‡ All studies utilized HLA serotyping, except that of Gibson *et al.*, (1999) which utilized genotyping

§ Total controls per group is not equal to total number of controls, because the same control groups were used by some investigators, [This table has been reproduced (with slight modification) by seeking permission from the Review , by Padraig Wright *et al.*, title “Schizophrenia and HLA: a review”, Volume 47, pg no.4-5, Copyright Elsevier, 2001.]

Past association studies with various Class I and Class II alleles yielded inconsistent results[14] except HLA-A*9 (now subdivided into A*23/A*24).[15] In different ethnic population, associations have been found for HLA-A9,[16] HLA-A23,[17] HLA-A*24,[18] HLA-A*1,[19] HLA-A*2, HLA-A*3, HLA-A*11, HLA-B*17, HLA-B*27, HLA-B*8 and Cw 2,[20] HLA-A*3.[21] The reason for the inconsistencies include the diagnostic methods, particularly in early studies , are imprecise and vary greatly.[22] The majority of the previous association studies were carried out using serological typing techniques [microlymphocytotoxicity testing][23], which have been found to be inaccurate, with 7-25% misassignment errors[24] compared with the DNA based techniques (polymerase chain reaction (PCR) and sequence specific oligonucleotide probes (SSOP). The source of controls is not always described in sufficient detail to ensure that results are not simply due to population stratification. Significant results are not always corrected for the number of statistical tests performed.[25]

The present study has been carried out to investigate the association of HLA Class I alleles in Schizophrenia with the help of DNA-based typing method in well-characterized sample of ethnically matched patients and controls. The study may help to identify disease-specific susceptibility (risk) and protective markers that can be used in immunogenetic profiling, risk assessment and therapeutic decisions. Further, the study may refine already known associations in the light of modern DNA based HLA typing method.

## MATERIALS AND METHODS

We studied 50 India-born schizophrenic patients residing in and around Siliguri subdivision of West Bengal, referred to the psychiatric outpatient department (OPD) of Psychiatry, North Bengal Medical College and Hospital. Three major selection criteria were considered for selection of schizophrenic group; (i) unrelatedness of individuals from each other, (ii) resident of the state of West Bengal and (iii) subjects satisfying DSM IV[3] diagnostic criteria for schizophrenia . The exclusion criteria followed in the present study include; (i) history of substance abuse, (ii) presence personality disorder, (iii) presence of dementia and mental retardation. The patients considered for the present study were belonging to the Bengali, Nepali, Bihari and some tribal community. They were diagnosed independently by two psychiatrists using Structured Clinical Interview[26] and according to the standard diagnostic criteria of DSM IV and assessed by the Brief Psychiatric Rating Scale (BPRS).[27] The present study comprise of 45 Paranoid, 2 Residual, 2 Undifferentiated and 1 disorganised schizophrenic patients. Considering the small number of different subtypes of schizophrenic patients in this study (except Paranoid), we have considered schizophrenic patients as a whole. Some of the demographic variables which have been studied in the patient group are given in the table 2.

**Table 2:** Psycho-socio-demographic characteristics of the schizophrenic patients

	Standard deviation		
Gender			
Male	P=0.78 (78%)		Z=6.49
Female	p<0.001		
Age			
Mean	34.06	9.46	
Disease duration (in years) mean	5.77	6.37	
Substance abuse			
Yes	P=0%		
No			
Marital status			
Married	P=0.64 (64%)		Z=2.06
Unmarried	p<0.05		
Ethnicity			
Bengali	68%		X^2^=51.28
Nepali	10%		(d.f.=3)
Tribal	18%		p<0.001
Bihari	4%		

A total number of 50 ethnically matched healthy individuals were considered as controls. To avoid the spurious associations resulting from population stratification great care was taken. The following criteria were strictly followed for the selection of controls, (i) same ethnic group as the patients, (ii) sex and age matched with patients, (iii) absence of family history of autoimmune or psychiatric disorder, (iv) recent history of intercurrent infection and allergies, (v) unrelatedness of individuals from one another, (vi) no history of any substance abuse. All the participants provided their written consent for giving the blood sample after the study procedures were explained.

### Methodology

The blood was drawn by vein puncture method and EDTA was added as anticoagulant. DNA was extracted from peripheral mononuclear cells of the blood by the Phenol Chloroform method. The typing of HLA Class I was performed by PCR-SSP technique. The typing and sequence information of primers were taken from Bunce *et al.*, (1995).[28] The primers, Taq polymerase, nucleotides etc. were obtained from Bangalore Genei, India. In general 25µl of reaction mixture include 1 × PCR buffer, 200µM of each of dNTP, 1.5mM MgCI2, 0.4µM of forward and reverse primers, 100ng of genomic DNA and 1unit of Taq polymerase. The amplifications were accomplished on a thermal cycler (Perkin Elmer, USA). PCR reaction are subjected to 30cycles, each consisted of 94°C for 30s, 60°C for 1min. and 72°C for 1min. with initial denaturation step of 2min and final extension of 2min.

### Statistical analysis

The phenotype frequencies were calculated by direct count. χ^2^ test was performed to compare the frequency of each antigen among the patient and control group followed by Fisher's exact test. Since testing for a large number of antigens can reveal at least one positive association where none really exists, the *p* values from each Fisher's exact test had to be less than the Bonferroni *p* [0.05 divided by the number of antigens tested which equals to 0.003125] to be called statistically significant.[21] Relative risk was estimated by using Haldane's method (1956).[29]

### RESULTS AND DISCUSSION

The incidence and frequency of HLA Class I antigens among patients and control has been presented in table 3. There was a significantly higher frequency of HLA-A*03 (χ^2^=11.458, *p*=1.155e-4) in patients than the control groups. On the other hand HLA-A*25 (χ^2^=13.619, *p*=9.185e-5), A*31 (χ^2^=22.562, *p*=8.793e-8) and B*51 (χ^2^=40.047, *p*=1.604e-12) showed lower value significantly even after the Bonferroni correction. Though A*02 (χ^2^=6.052, *p*=6.699e-3)showed lower frequency and B*07 (χ^2^=4.069, *p*=2.035e-2) and B*42 (χ^2^=4.522, *p*=1.632e-2) showed higher frequency they were not found to be significant after the Bonferroni correction.

**Table 3:** Phenotype frequency, Chi square, relative risk (RR) values and probability of HLA-A and B loci alleles in the patients with schizophrenia and healthy controls

Antigen	Patients (N=50)	Control (N=50)	Chi-square	Chi square (y)	RR	*P* value
A*02	24	37	7.103	6.052	0.332	6.699e-3*
A*03	50	38	13.636	11.458	32.792	1.155e-4†
A*11	36	31	1.130	0.723	1.558	1.975e-1
A*23	21	26	1.003	0.642	0.673	2.115e-1
A*24	32	26	1.477	1.026	1.624	1.555e-1
A*25	10	29	15.174	13.619	0.188	9.185e-5†
A*26	21	17	0.679	0.382	1.395	2.684e-1
A*29	33	32	0.043	0.000	1.089	5.000e-1
A*30	33	25	2.627	2.011	1.914	7.787e-2
A*31	0	20	25.000	22.562	0.014	8.793e-8†
B*07	47	39	5.315	4.069	3.951	2.035e-2*
B*21	36	38	0.207	0.051	0.817	4.099e-1
B*4001	17	13	0.761	0.428	1.451	2.565e-1
B*4201	39	28	5.472	4.522	2.711	1.632e-2*
B*44	14	10	0.877	0.493	1.532	2.414e-1
B*5101-5105	0	30	42.857	40.047	0.006	1.604e-12†

y= Yates's correction

* Significant

† Significant after the Bonferroni correction Bonferroni's probability is 0.003125

Note= The abbreviation 1.156 e-4 means 1.156 × 10-4, like wise the other values may be interpreted.

A significant higher frequency of HLA-A*03 observed in the present study is in accordance with the previously reported study by Debnath *et al.*[21] which is also in accordance with the study of Rudduck *et al.*, (1984a, 1984b)[30] in Swedish population. Although the association was found to be significantly higher, the present study did not reveal very strong association as it has been reported earlier.

On the other hand, in the present study A*25, A*31 and B*51 showed significantly lower negative value which is the unique finding of the present study. Among these alleles, A*31 and B*51 showed strong negative associations (RR=0.014 and RR=0.006 respectively). The increased frequency of A*11 found in the previous study was not reproducible in the present study. Apart from this, several other alleles like B*7 and B*42 showed higher value but were not statistically significant. We also observed a negative association of A*2 but the association was not found to be significant which was in accordance with the findings of Debnath *et al.*[21] But the finding was unlike the previous findings by Luchins *et al.*, (1980)[31] which showed positive association of A*02 with schizophrenia in African-USA population. However, we have not found association between HLA-A*23 and A*24 in our study as has been reported by previous studies.

Many microbial factors have been implicated in the pathogenesis of schizophrenia, but so far each microbial factor has been identified in a relatively small subgroup of patients.[32][33] The heterogeneity of these microbial factors is also reflected by the associations with different HLA loci and their alleles.[34] Polymorphic HLA molecules process, select and present degraded microbial proteins.[35][36] The set of inherited HLA alleles determines susceptibility or resistance to particular microbes.[34]

The analysis of the demographic variables suggests the present schizophrenic population is not in equal composition for the different ethnic group. The study comprises more number of Bengali populations. However, as mentioned earlier they were strictly matched according to their ethnicity, age and sex with the patients. The study comprise of higher number of male schizophrenics, which suggest the higher vulnerability of men to this disorder, at least in this region. The higher number of married patients in this study may be due to the strict social customs and strong social bondage of the Indian society.

The present study supports earlier finding of association of HLA-A*03 with schizophrenia along with the negative association of some more alleles, which is the new finding of the present study. However, it is too early to speculate the exact mechanism of the association. The result is preliminary and so far not correlated with the parameters like birth status, viral infections, prenatal infections, etc. However, this study provides the evidence for the possible existence of a susceptibility locus for schizophrenia within the HLA region. Given the size of our sample the result of our finding should be interpreted with caution. The present study needs to be replicated in the large sample size to strengthen our hypothesis of genetic association of HLA Class I antigen with schizophrenia.
